# The science at HIVR4P 2024: The era of choice in biomedical HIV prevention

**DOI:** 10.1002/jia2.70001

**Published:** 2025-07-01

**Authors:** Beatriz Grinsztejn, Victor Appay, Linda‐Gail Bekker, Chris Beyrer, Deborah Donnell, Jorge Sanchez, Davina Canagasabey, Carolina Coutinho, Yonatan Ganor, Vincent Muturi‐Kioi, Katrina F. Ortblad, Erin Cooney, Gastón Devisich, Paula Ellenberg, Yanina Ghiglione, Kevin K'Orimba, Phionah Kibalama Ssemambo, Natasha Tatiana Ludwig‐Barron, Dieter Kenneth Mielke, Ranajoy Mullick, Michelle Kathini Muthui, Pablo D. Radusky, Emmanuel Sendaula, Syed Raza Haider Tirmizi, Akemi V. Matsuno Sanchez, Julian Vega, Roger Pebody

**Affiliations:** ^1^ Fundação Oswaldo Cruz (Fiocruz) Rio de Janeiro Brazil; ^2^ Université de Bordeaux Bordeaux France; ^3^ Desmond Tutu HIV Centre Cape Town South Africa; ^4^ Duke Global Health Institute Durham North Carolina USA; ^5^ The Fred Hutchinson Cancer Center Seattle Washington USA; ^6^ Centro de Investigaciones Tecnológicas Biomédicas y Medioambientales, Universidad Nacional Mayor de San Marcos Lima Perú; ^7^ Primary Health Care Program PATH Washington DC USA; ^8^ Université Paris Cité, Institut Cochin Paris France; ^9^ IAVI Nairobi Kenya; ^10^ Public Health Sciences Division Fred Hutchinson Cancer Center Seattle Washington USA; ^11^ Department of International Health Johns Hopkins Bloomberg School of Public Health Baltimore Maryland USA; ^12^ Research Department Fundación Huésped Buenos Aires Argentina; ^13^ Life Sciences, Burnet Institute Melbourne Victoria Australia; ^14^ CONICET – Universidad de Buenos Aires, Instituto de Investigaciones Biomédicas en Retrovirus y SIDA (INBIRS) Buenos Aires Argentina; ^15^ MOSAIC, LVCT Health Nairobi Kenya; ^16^ IAVI New York USA; ^17^ Makerere University Johns Hopkins University (MU‐JHU) Research Collaboration Kampala Uganda; ^18^ Division of Prevention Science, Department of Medicine University of California San Francisco California USA; ^19^ Department of Surgery Duke University Durham North Carolina USA; ^20^ Antibody Translational Research Program, Translational Health Science and Technology Institute, Virus Research, Therapeutics and Vaccines Faridabad India; ^21^ IAVI New Delhi India; ^22^ Biosciences Department KEMRI‐Wellcome Trust Research Programme Kilifi Kenya; ^23^ Faculty of Psychology Universidad de Buenos Aires Buenos Aires Argentina; ^24^ Monitoring and Evaluation, Reach Out Mbuya Community Health Initiative Kampala Uganda; ^25^ Dostana Male Health Society Lahore Pakistan; ^26^ Hospital General de Agudos “Juan A. Fernández” Buenos Aires Argentina; ^27^ IAS Consultant Paris France

**Keywords:** client choice, HIV prevention, implementation science, pre‐exposure prophylaxis, post‐exposure prophylaxis, broadly neutralizing antibodies, vaccines

## Abstract

**Introduction:**

HIVR4P 2024, the 5th HIV Research for Prevention Conference, took place in Lima, Peru, 6–10 October 2024. The conference focused on new developments in HIV prevention from basic research to new product development and implementation science.

**Methods:**

Sessions were assigned to one of five tracks: basic science; pre‐exposure prophylaxis (PrEP) and antiretroviral (ARV)‐based prevention; vaccines and broadly neutralizing antibodies (bNAbs); applied and implementation science; and other prevention modalities and cross‐cutting issues. A team of rapporteurs covered each track and identified conference highlights.

**Results:**

Strategies to elicit bNAb responses by vaccination are advancing to clinical trials, while combination bNAbs show promise as an alternative to ARV‐based products. There is promising diversity in the PrEP product pipeline and twice‐yearly lenacapavir has demonstrated exceptional efficacy, but barriers to widespread access and implementation remain, compounded by new challenges from the significant policy changes and funding reductions of the new US administration. Innovative ways of delivering PrEP to vulnerable communities that could benefit are being explored and, in some cases, have been successfully implemented.

**Discussion:**

Choice in HIV prevention products and differentiated delivery models that enable clients to select options that meet their preferences and changing needs is essential. Additionally, the involvement of the community throughout the design, implementation and dissemination process is necessary to maximize the impact of HIV prevention. Ensuring equitable access in a rapidly changing context will involve policy changes, partnerships with local organizations and addressing social determinants that impact health outcomes.

**Conclusions:**

We are in an era with more tools than ever before to prevent HIV acquisition; now, we need to facilitate collaborations between diverse stakeholders, including researchers, community members, policymakers, healthcare providers and funders. The future of HIV prevention should lie in a holistic approach that respects individual choice, enhances service accessibility and is flexible to meet evolving challenges and opportunities. However, policy changes since the conference ended have profoundly altered the HIV prevention landscape and threaten the advances described in this report.

## 1 INTRODUCTION

Despite considerable advances in HIV prevention and treatment over recent decades, ∼1.3 million (1–1.7 million) people acquired HIV in 2023 [1], far above UNAIDS’ goal to reduce the number of new acquisitions to <370,000/year globally by 2025 [2]. Promising new antiretroviral (ARV)‐based HIV prevention products such as long‐acting cabotegravir (CAB‐LA) [3] are in development or are becoming available, but barriers to implementation of, and widespread access to, these products remain [2]. While the Antibody Mediated Prevention (AMP) trials provided proof of concept that prophylaxis with broadly neutralizing antibodies (bnAbs) can be effective [4] and have informed vaccine strategies aimed at inducing such antibodies, the translation of these findings into accessible preventive tools remains a complex challenge that demands sustained investment and scientific innovation.

HIVR4P 2024, the 5th HIV Research for Prevention Conference, is the only global scientific conference exclusively dedicated to the rapidly evolving field of HIV prevention research [5]. Other leading HIV meetings, such as the International AIDS Conference and the Conference on Retroviruses and Opportunistic Infections (CROI), include HIV prevention alongside treatment, co‐morbidities and cure. During the conference, which was held in Latin America for the first time, the scientific community addressed challenges and opportunities in HIV prevention research and implementation. More than 1300 people from more than 50 countries participated in person or virtually, including 203 scholarship recipients and 21 journalist fellows. This paper aims to review the highlights of the conference.

## 2 METHODS

Sessions were assigned to one of five tracks: basic science; pre‐exposure prophylaxis (PrEP) and ARV‐based prevention; vaccines and bNAbs; applied and implementation science; and other prevention modalities and cross‐cutting issues.

A team of rapporteurs reviewed and identified the highlights of each track. For each track, a lead rapporteur was nominated by the conference Organizing Committee and confirmed by the conference Co‐Chairs. Each lead rapporteur subsequently selected three rapporteurs, inviting participation from scholarship recipients with accepted abstracts at the conference. Expertise relevant to the track as well as regional and gender balance were considered in the selection.

This manuscript is based on the rapporteurs’ session reports and insights. For each track, approximately 10 studies or presentations were selected as highlights, based on the following criteria: studies with significant implications for the field, research that reflected prominent themes or concerns of the conference, and particularly innovative work.

## 3 RESULTS

### 3.1 Basic science

The Basic science track covered recent research that provides the scientific rationale for new developments in HIV prevention (Table 1). Interactions between HIV and the microbiota in the mucosal epithelia of the vagina, gastrointestinal tract and foreskin impact HIV/simian immunodeficiency virus (SIV) acquisition. In non‐human primates (NHPs), microbiome dysbiosis influences rectal SIV acquisition: NHPs with a microbiome disrupted by vancomycin had low‐level gastrointestinal (GI) tract inflammation and greater numbers of transmitted/founder variants than those with healthy microbiomes. However, dysbiosis did not accelerate disease progression [6]. In a second study, intravaginal inoculation of humanized mice with female genital tract bacteria provided a novel *in vivo* model for investigating genital inflammation and HIV risk in women. Intravaginal inoculation of pro‐inflammatory *Prevotella bivia* resulted in an increased risk of HIV acquisition compared to the risk in mice inoculated with *Lactobacillus crispatus*. This was attributed to elevated numbers of antigen‐presenting cells and higher inflammatory cytokine levels. A possible next step for the research is to determine if maintaining a healthy microbiota with optimal *L. crispatus/P. bivia* abundance could reduce HIV acquisition in female humanized mice *in vivo* [7]. A study randomized men to one of four antimicrobials prior to voluntary male medical circumcision, or to immediate circumcision, in order to investigate how antimicrobials impact penile immunology and HIV susceptibility. Topical metronidazole and oral tinidazole decreased HIV entry into foreskin CD4^+^ T cells *ex vivo*; topical metronidazole and topical clindamycin had major impacts on dysbiotic bacteria and other CD4^+^ T cell targets. This suggests that clinical interventions targeting the penile microbiota (antibiotics, probiotics) could be explored [8].

**Table 1:** Highlights of the basic science track

Gastrointestinal microbiome	Microbiome dysbiosis in primates increased transmitted/founder variants in rectal SIV acquisition [6].
Vaginal microbiome	*P. bivia* increased HIV acquisition in humanized mice versus *L. crispatus* by elevating inflammatory cells and cytokines [7].
Penile microbiome	Antimicrobials decreased HIV entry into human foreskin CD4^+^ T cells *ex vivo* [8].
CD8^+^ T cell control	Low CD8^+^ T cell avidity may explain vaccine failures; improved vectors could enhance killing capacity [9].
HLA‐E‐restricted CD8^+^ T cells	HLA‐E/HIV peptides trigger T cell killing *in vitro* [10].
Pro‐inflammatory macrophages	CX3CR1^+^ macrophages accumulated in SIV‐infected macaques during an ATI; restoration was associated with post‐treatment control [11].
SIV reservoirs	Reservoirs in colon tertiary lymphoid organs showed low translation status favouring viral persistence [12].
IL‐10 signalling	IL‐10 neutralization plus PD‐1 blockade reduced viral reservoirs and enabled post‐treatment viral control [13].
Monocyte gene expression	Increased monocyte IL‐1 with higher central memory CD4^+^ T cells predicted smaller reservoirs [14].
Complete reservoir clearance	ART, leronlimab and bNAbs prevented viral rebound in infant macaques, suggesting early reservoir clearance [15].

Data presented on CD8^+^ T cell control of HIV suggested novel potential approaches that should be investigated. One of the reasons for the failure of early vaccine designs may be that induced CD8^+^ T cells have low functional avidity that is insufficient to respond to the low levels of antigen on an HIV‐infected CD4^+^ T cell. Therefore, perforin accumulation that requires prolonged stimulation, and subsequent maximal degranulation, are not achieved. Low‐avidity responses might benefit from further clonal selection with heterologous or persisting vectors, with the killing of HIV‐infected CD4^+^ T cells as a benchmark [9]. Data were presented on HLA‐E‐restricted HIV‐specific CD8^+^ T cell responses. Such cells are rare in natural HIV infection due to the low presentation of HIV peptides by HLA‐E and inefficient priming, but priming has been achieved *in vitro*. It was shown that HLA‐E/HIV peptides on the surface of infected cells are sufficient to trigger T cells to kill targets. If these cells could be primed *in vivo*, they might become a universal immunotherapeutic intervention [10]. In SIV‐infected macaques who received either early or late antiretroviral therapy (ART) followed by an analytical treatment interruption (ATI), analysis of mucosal specimens revealed a notable accumulation of pro‐inflammatory CX3CR1^+^ macrophages, which positively correlated with viral load levels. Animals classified as post‐treatment controllers exhibited restoration of macrophage homeostasis, suggesting a potential role in viral control post‐therapy [11]. It now needs to be determined whether modulating the myeloid compartment could have benefits in the context of HIV persistence and control after ATI. Future studies should determine the precise roles of different monocyte and macrophage subsets.

Turning to HIV‐1 reservoirs, findings from spatial transcriptomics and immunofluorescence assays were used to characterize the microenvironment of SIV tissue reservoirs. Persistent reservoirs were associated with tertiary lymphoid organs in the colon and might be characterized by a status of low translation consistent with stress responses. This status could be favouring long‐term viral persistence during ART and rapid rebound of robust viral populations post‐ATI [12]. Data from NHPs showed that IL‐10 is upregulated in the neighbourhood of infected cells. Before and during ART, IL‐10 expression is in close spatial proximity to latent and active viral reservoirs, suggesting that IL‐10 signalling aids in maintaining viral reservoirs *in vivo*. IL‐10 neutralization reduced viral reservoirs within B cell follicles and the expression of genes associated with pathways of cell survival, B cell differentiation and T follicular helper (TFH) cell homeostasis. Neutralization of IL‐10 and blockade of PD‐1 led to significant viral control for >24 weeks after an ATI [13]. A study using unbiased transcriptomics that screens all RNA of a human cell showed that in samples of people living with HIV (PLWH) at Fiebig stage 3, monocyte gene expression profiles were associated with reservoir size. Increased expression of IL‐1β in monocytes in the presence of higher frequencies of central memory CD4^+^ T cells predicted a smaller reservoir, with findings validated in an independent cohort of PLWH with different genetic backgrounds and a different HIV‐1 subtype, treated during acute HIV infection. This effect could involve IL‐1β‐mediated NFkB activation in infected cells [14].

Finally, there were impressive results indicating complete reservoir clearance in infant simian‐human immunodeficiency virus (SHIV)‐infected macaques. Beginning 72 hours after infection with SHIV, eight animals received ARV therapy; leronlimab, a monoclonal IgG4 antibody that targets CCR5; and two bNAbs (PGT121‐M428L/N434S (LS) and VRC07‐523‐LS). Following ATI, no rebound, cell‐associated viral DNA or viral DNA in lymph nodes was found, even following CD8^+^ T cell depletion. The results suggest that the combination of ART, bNAbs and CCR5 blockade via leronlimab synergize in an undefined mechanism to prevent further seeding of the reservoir in the early post‐infection period, and may have permanently reduced its size. Whether this effect could be explained by previous findings showing that the CCR5 blocker maraviroc acts as latency reversing agent (LRA) remains to be determined. This may be a promising strategy for cure from HIV in infant populations acquiring HIV through vertical transmission, and should also be tested in adults [15].

### 3.2 Vaccines and bNAbs

Efforts to develop HIV vaccines are currently focused on eliciting bNAbs (Table 2). Although previous HIV vaccine trials failed to show protection, they provided useful lessons that have informed the design of new‐generation immunogens. A rational approach to vaccine design is to identify a correlate of protection and then to create a strategy that can elicit it. The phase 1 IAVI‐G003 study provided proof of concept of the germline targeting strategy, which involves the selection and sequential maturation of rare precursors to generate broad protective responses. However, designing and testing this maturation path will take time and concerted effort [16].

**Table 2:** Highlights of the vaccines and bNAbs track

Germline targeting	The phase 1 IAVI‐G003 study provided proof of concept for generating protective antibody responses [16].
Mutation‐guided design	Prime‐boost immunogens can select critical mutations and induce antibodies with neutralization breadth in mice [17].
Vaccine dosing strategy	Fractional escalating doses enhanced immune responses compared to single‐dose strategy for germline‐targeting vaccines [18].
Adjuvant comparison	3M‐052‐AF + Alum adjuvant elicited broad ADCC and neutralizing responses in trial comparing adjuvants [19].
bNAb precursors in Kenya	VRC01‐class bNAb precursors exist at similar frequencies to previous studies despite genetic diversity [20].
bNAb advantages	Long‐acting bNAbs could offer 6‐month protection with different resistance patterns than ARVs, and may have a particular role in preventing vertical transmission [21, 22].
Triple bNAb combinations	Triple combination could cover most global HIV‐1 strains with 6‐month dosing [23]. Three enhanced bNAbs protected against mixed SHIV challenges better than single antibodies [24].
Co‐administration of bNAbs	No pharmacokinetic interference observed when VRC07‐523LS was co‐administered with other bNAbs [25].
Evolution of resistance	Recent HIV viruses show significantly higher resistance to antibody‐dependent cellular cytotoxicity than historic strains [26].

Functional, improbable mutations are a rate‐limiting step in eliciting bnAbs. A mutation‐guided vaccine design strategy aimed to accelerate bNAb induction by targeting selection to these rate‐limiting steps. Results from preclinical studies in knock‐in mice demonstrated that prime‐boost immunogens can select critical improbable mutations in bNAb B cell lineages and induce antibodies with neutralization breadth after bNAb precursor expansion. The strategy is now being tested in humans [17]. In the HVTN 301 study, a fractional escalating dose strategy was compared with a bolus (single dose) strategy for a novel germline‐targeting HIV vaccine (426.mod.core‐C4b). Fractional escalating doses were safe, tolerable and enhanced antibody, B cell, and CD4^+^ T cell responses during the priming phase (approximately 35% of participants developed VRCO1‐class B cell responses compared to 9% in the bolus group). HVTN 301 established proof of concept that sustained antigen/adjuvant exposure can improve immune responses for preventive HIV vaccines intended to elicit bNAbs during the priming phase [18]. While adjuvants play a significant role in the quality of immune responses elicited by a vaccine, direct comparisons of adjuvants with the same immunogen are rare. A clinical trial compared three adjuvants and found that 3M‐052‐AF (i.e. a TLR7/8 agonist) + Alum represents a potent adjuvant that can elicit broad antibody‐dependent cellular cytotoxicity (ADCC) responses in addition to neutralizing responses. It represents a strong candidate for use in future HIV vaccine trials [19].

The success of germline targeting relies on the composition of the naive B cell repertoire, but this is insufficiently characterized, particularly in African populations. Researchers, therefore, collected peripheral blood mononuclear cells (PBMCs) from 60 participants in three Kenyan regions and assessed bNAb precursor frequencies. They found that despite genetic diversity in the naive B cell repertoire and varied environmental exposure (e.g. to malaria), bNAb precursors of the VRC01 class are present at similar frequencies to previous studies. This supports evaluating the germline targeting vaccine strategy for VRC01‐class bNAb induction within this population [20].

In a context in which two long‐acting ARV‐based PrEP products have already been proven to be highly effective, and in which others are in development, a symposium speaker made the case that bNAb‐based prevention still has a role to play, has potential advantages and could offer additional HIV prevention options. With structural modification, monoclonal antibodies may have great breadth and potency, and could potentially be administered to adults every 6 months via intravenous infusions. They appear to have favourable safety and pharmacokinetic (PK) profiles, and as they have a different mechanism of protection to ARVs, they could mitigate the risk of resistance to ARVs and be used to cover the “long tail” after cessation of injections [21]. They may also have a particular role in preventing vertical transmission during breastfeeding. However, several challenges remain regarding the design and conduct of efficacy trials in this population [22].

Following the efficacy signal seen in the AMP trials, bNAbs against HIV have been tested in multiple clinical trials. The available evidence suggests that a combination of bNAbs could be tested in an efficacy trial, for example the triple combination of VRC07‐523LS, PGT121LS and PGDM1400LS, which is expected to cover most global HIV‐1 strains. Moreover, desired LS mutations extend the antibody half‐life and support the dosing of this combination every 6 months [23]. In rhesus macaques, a cocktail of three enhanced bNAbs (ePGT121v1, ePGDM1400v9 and VRC01.23J1), engineered for increased half‐life and improved neutralization breadth and potency, conferred *in vivo* protection against repeated high‐dose mixed SHIV challenges. Animals receiving one bNAb became more rapidly infected when challenged with multiple SHIVs than with one SHIV, while the bNAb cocktail delayed infection from exposure to diverse isolates [24]. An analysis combined data from three trials of the broadly neutralizing monoclonal antibody VRC07‐523LS in order to evaluate PK features and demonstrated there was no interference with the PK profile when VRC07‐523LS was co‐administered with other bNAbs [25].

HIV diversity represents a significant barrier to effective monoclonal antibodies, and a study explored change over time, using a panel of historic versus recently circulating Envelope sequences. The proportion of recent viruses resistant to ADCC mediated by multiple monoclonal antibodies was significantly higher than historic viruses. Two sites in the HIV Envelope were associated with increased resistance to ADCC and have increased in frequency over time [26].

### 3.3 PrEP and ARV‐based prevention

A highlight of HIVR4P 2024 (Table 3) was the efficacy results for long‐acting lenacapavir as PrEP in cisgender gay, bisexual, and other men, transgender women, transgender men and gender non‐binary persons who have sex with partners assigned male at birth. Researchers adopted an intentional approach to enrol historically underrepresented populations and the PURPOSE 2 study was celebrated as “the most racially, ethnically, and gender‐diverse PrEP trial to date.” Of 3273 randomized participants, 67% were non‐White, 63% Hispanic/Latine, 15% transgender women and 6% gender non‐binary. The phase‐III, double‐blinded, randomized controlled trial evaluated the efficacy of twice‐yearly lenacapavir injections to prevent HIV compared to background HIV incidence (primary endpoint) and daily, oral emtricitabine/tenofovir disoproxil fumarate (F/TDF, secondary endpoint). Twice‐yearly lenacapavir significantly reduced HIV incidence by 96% compared with background HIV incidence, and by 89% compared with F/TDF. It is hoped that twice‐yearly lenacapavir will increase PrEP uptake, adherence and persistence in populations disproportionately affected by HIV [27, 28], but this will require structural and systemic barriers to access to be addressed.

**Table 3:** Highlights of PrEP and ARV‐based prevention track

Six‐monthly lenacapavir injections	Lenacapavir reduced HIV incidence by 96% versus background incidence in the diverse PURPOSE 2 study [27, 28].
Dapivirine vaginal ring	Three‐month 100 mg ring showed pharmacokinetic superiority over licensed monthly 25 mg ring [29]. Following the use of monthly ring during early pregnancy, there were no adverse effects on pregnancy or infant outcomes [30].
CAB‐LA and contraception	No significant interactions found between long‐acting cabotegravir and contraceptives [31].
Injectable hydrogel depot platform	Animal model suggests the platform could deliver HIV prevention and contraception simultaneously [32].
Transcutaneously refillable implant	The implant provided 29‐month islatravir release with 100% efficacy against SHIV in primates [33].
Tenofovir rectal douche	A douche would be acceptable and behaviourally congruent for young gay and bisexual men [34].
DoxyPEP	Moderate uptake but large reduction in STIs among users in Italy [35].
Engaging youth	Youth‐friendly services involve meeting young people in their spaces with appropriate language [36].

A late‐breaking abstract described another innovative HIV prevention product: a 100 mg dapivirine vaginal ring (DVR) designed to be replaced every 3 months. A phase I, open‐label, randomized, crossover trial investigated the relative bioavailability of the 100 mg DVR to the licensed 25 mg DVR, which is replaced every 1 month. The study demonstrated the PK superiority of the 3‐month ring, with only modest increases in overall drug exposure, indicating that the 3‐month ring should have at least equal efficacy to the 1‐month ring. Regulatory approval will be sought as a line extension [29].

Two studies provided data on the use of PrEP products during pregnancy or with concurrent use of contraceptives. MTN‐025/HOPE open‐label extension findings showed no notable adverse effects on pregnancy or infant outcomes due to DVR use during early pregnancy. This study adds to the evidence supporting the safety of ring use throughout pregnancy [30]. A sub‐study of HPTN 084 assessed potential pharmacologic interactions between PrEP agents (CAB‐LA or TDF/FTC) and contraceptives (etonogestrel, medroxyprogesterone acetate or norethindrone). No significant interactions were observed with CAB‐LA, indicating that co‐administration is possible, but data on TDF/FTC was inconclusive due to low adherence [31].

Several abstracts reported on early studies of innovative prevention products that may eventually offer clients greater PrEP choice. A subcutaneous injectable hydrogel depot platform that provides lower injection volume and higher drug loading capacity allows co‐delivery of two drugs with discretely controlled release profiles, and could, therefore, meet the need for simultaneous protection against HIV acquisition and unintended pregnancy. Formulations containing cabotegravir or dolutegravir alone and combined with levonorgestrel were administered to rats in order to assess PK, drug−drug interactions and injection site reactions for up to 3 months [32]. A drug‐agnostic, transcutaneously refillable subdermal implant provided ultra‐long‐acting HIV prevention through the sustained release of islatravir (up to 29 months) and showed 100% efficacy against SHIV in an NHP study. The researchers also reported limited animal data on MK‐8527 and human studies with three other ARVs are planned [33]. As research with potential end‐users on acceptability and product preferences is needed throughout the product development continuum, the ATN 163 study reported on the acceptability of a novel HIV prevention douche among gay, bisexual and other men who have sex with men (GBMSM) aged 18−24 in the United States. Douching before receptive anal intercourse is a common practice in this population; an on‐demand rectal douche containing TFV for PrEP was found to be highly acceptable and behaviourally congruent [34].

A retrospective analysis reported on the real‐world impact of doxycycline post‐exposure prophylaxis (DoxyPEP) among GBMSM in Milan, Italy. The uptake of doxyPEP was moderate (29.4% of those counselled). Among doxyPEP users, there was an 80% reduction in chlamydia, syphilis and gonorrhoea incidence, in comparison with the time period before receiving doxyPEP [35].

Discussing the barriers adolescents face when engaging with prevention services in Brazil, a speaker shared their experience creating youth‐friendly services that prioritize the unique needs of young people. Whether through face‐to‐face contact or social media platforms, it was emphasized that demand cannot be generated if you do not interact in the spaces where young people are present and use appropriate language [36].

### 3.4 Applied and implementation science

Table 4 provides a summary of track highlights. Much of the programmatic data on PrEP focuses on the number of clients who initiate PrEP, with limited information available on persistence and other long‐term outcomes. However, data from PrEP clients who returned for visits after initiation in 51 PEPFAR‐supported countries (the analysis does not include clients who do not return to the facility) indicates that between 0.14% and 0.23% were diagnosed with HIV per quarter. This suggests that most returning clients were using PrEP effectively [37]. However, most PrEP programmes globally are not sufficiently scaled‐up. Based on HIV incidence rates, PrEP initiations and the volume of PrEP distributed, researchers classified 77 countries into five typologies. They found that focusing on “growing and high volume” countries—that is those where HIV incidence is high and PrEP delivery systems are in place but not yet scaled up—could have the greatest impact on incidence. In each of these countries—Brazil, Mozambique, Nigeria, South Africa, Tanzania and Uganda—CAB‐LA has already been approved, and in two, the DVR is also approved [38].

**Table 4:** Highlights of the applied and implementation science track

PrEP use in PEPFAR	Only 0.14−0.23% of returning PrEP clients tested HIV positive quarterly, suggesting effective use [37].
Global PrEP scale‐up	The greatest epidemiological impact could be achieved by focusing on high‐incidence countries with existing PrEP systems [38].
Offering a choice of PrEP products	In two African studies, a majority of women initially chose oral PrEP, while others preferred the vaginal ring [39, 41]. Health facilities provided appropriate client counselling [40].
Men's preferences for long‐acting PrEP	South African men equally preferred implants and injections, but implants showed higher persistence rates [42].
Services for transgender women	Integrated PrEP and hormone therapy with peer navigation equalled the outcomes of the standard of care [43].
CAB‐LA implementation	User‐centred strategies overcame barriers to CAB‐LA integration in the United States [44]. Over 90% of CAB‐LA recipients in Zambia returned for their second injection [45].
Self‐assessment of HIV vulnerability	STI self‐testing at home increased PrEP restarts among adolescent girls who had discontinued [46].
Risk‐based language	Speaker advocated replacing risk‐based prevention with sex‐positive approaches [47].
Artificial intelligence	AI shows promise for HIV prevention but requires proper governance and oversight [48].

Now that more than one PrEP product is available in some settings, studies are focusing on offering clients a choice of PrEP products and the ability to switch between them as their circumstances or preferences change. The CATALYST study is assessing an enhanced service delivery package for informed PrEP choice for women in five African countries. In an interim analysis, 66% of women initially chose TDF/FTC and 34% initially chose the DVR. Younger women, new PrEP users, and pregnant and breastfeeding women were less likely to use the DVR, while those with more than one partner and those using contraceptives were more likely to choose the DVR [39]. Client surveys in CATALYST indicated that 87% had received appropriate choice counselling, suggesting that health facilities can offer clients choice [40]. In a second study among women aged 15–24 years old buying contraceptives at retail pharmacies in Kenya, 79% chose TDF/FTC and 21% the DVR. Providing multiple HIV choice and pregnancy prevention options at pharmacies may increase coverage for HIV PrEP and contraception [41].

There is less research on preferences for different PrEP modalities among men, but a clinical crossover study randomized 186 men (including 84 GBMSM) in South Africa to receive one placebo implant for 6 months and bimonthly placebo injections for 6 months. Implants and injections were highly acceptable and equally preferred, but persistence was greater for implants than injectables. This reinforces the importance of offering a choice of PrEP products and underscores the impact product characteristics have on uptake and persistence [42]. In a randomized‐controlled study conducted with transgender women in the United States and Brazil, the intervention arm received integrated provision of oral PrEP and gender‐affirming hormone therapy, along with strengths‐based peer health navigation. However, PrEP uptake and adherence were not superior to the standard of care arm. PrEP engagement was high among all participants, attributed to the supportive, affirming clinical environments in which the study was conducted [43].

Several research teams reported on early experiences with CAB‐LA, highlighting implementation challenges and successes. The PILLAR study evaluated strategies used in 17 PrEP clinics across the United States. The team overcame barriers to CAB‐LA integration through user‐centred strategies including flexible scheduling and drop‐in appointments, telehealth visits, and integrated care for comorbidities. Provider support (sufficient staffing, task sharing, continuous training, insurance authorization support, clinic/pharmacy coordination, organizing scheduling and re‐scheduling) optimized CAB‐LA delivery [44]. A comparable level of CAB‐LA continuation was demonstrated in a demonstration study in Zambia. Of the 609 people initiating CAB‐LA, 55.8% were female and 70% had not previously taken oral PrEP, suggesting that CAB‐LA has the potential to reach new clients not currently engaged in HIV prevention services. Among those eligible for a second CAB‐LA injection, 91% continued and only 3.9% stopped, primarily due to hepatitis B infection. However, these are early data; 33% of participants were not yet eligible for their second injection at the time of analysis [45].

Clients may sometimes discontinue PrEP due to low perceived HIV vulnerability. A small, randomized study provided tools which could help adolescent girls and young women (AGYW) aged 16–18 who had discontinued PrEP in South Africa to assess their own HIV vulnerability and potentially trigger PrEP restart. Participants in both study arms had access to self‐completion behavioural assessments via a phone app and those in the intervention arm also received at‐home sexually transmitted infection (STI) self‐testing kits. There were more PrEP restarts in the latter group, suggesting that home‐based STI self‐testing gives AGYW a clearer picture of vulnerability to HIV. Of note, STI incidence was high in this cohort (63.6% chlamydia and 21.8% gonorrhoea at 6 months), demonstrating the value of diagnostic testing to identify asymptomatic STIs [46]. Risk assessment was a key element in the previous study, and in HIV research and public health practice more generally. However, a thought‐provoking presentation called for a shift away from risk‐based HIV prevention. It was argued that the use of risk‐based language during assessments of PrEP eligibility exacerbates stigma. The speaker advocated for a sex‐positive approach that focuses on autonomy, benefits, pleasure and sexual wellbeing [47].

A symposium discussed the role artificial intelligence (AI) could play in supporting the delivery of HIV prevention services. AI chatbots can provide high‐quality, empathetic responses to patient questions and concerns, but they need to be trained on locally relevant data and human oversight is necessary to ensure the quality and accuracy of responses. AI models have been trained on the behavioural, demographic and geographic data of individuals newly diagnosed with HIV in order to focus on HIV testing recommendations. However, AI requires proper governance, to ensure clients’ autonomy/privacy, to protect users from harms caused by errors and to protect users’ health data [48].

### 3.5 Other prevention modalities and cross‐cutting issues

Track highlights (Table 5) include a genomic analysis of cohort data in rural KwaZulu, South Africa, which may challenge the hypothesis that age‐disparate relationships are driving HIV transmission among young women. Cluster analysis of 1049 HIV genomes produced 73 possible transmission linkages, with younger people over‐represented in the pairs. Women under 30 tended to pair with men older than them, whereas women over 30 tended to pair with men the same age or younger than them. The researchers concluded that the observed age disparity may be explained by the population structure of the transmission network. There is an increased proportion of 25‐ to 29‐year‐old women and 30‐ to 34‐year‐old men in the transmission network compared to the total population of people living with HIV in the cohort. The authors suggest that this may reflect more recent transmission in these age groups [49]. The study demonstrates the potential of HIV molecular epidemiology to guide public health interventions, as explored in a symposium. These allow clusters of new infections to be rapidly identified, allowing focussed testing and linkage to care interventions to be offered, and for programming to be prioritized for groups who are more likely to be involved in HIV transmission and acquisition [50]. However, ethical concerns about privacy, human rights and transparency must be managed carefully. In the United States, there is a lack of informed consent and transparency about the use of individuals’ clinical data for molecular surveillance, and concern about increased risks of prosecution and stigmatization of groups [51].

**Table 5:** Highlights of the other prevention modalities and cross‐cutting issues track

Age disparities	A genomic analysis suggests that population structure, not age disparities, drives HIV spread among young women in South Africa [49].
Molecular epidemiology	Molecular surveillance can guide targeted interventions but raises ethical concerns about privacy, stigmatization and criminalization [50, 51].
Digital health interventions	Teleconsultations and flexible medication pickup locations increased access to PEP and PrEP in Brazil [52]. A phone app enhanced testing access and PrEP adherence in Malaysia [53].
Expedited STI therapy	Over 90% of young women in South Africa successfully delivered expedited therapy to partners, reducing STI recurrence rates [54].
Ugandan Anti‐Homosexuality Act	Local partners provided emergency social and legal support [55].
Adolescent PrEP policies	Only 19 of 29 African countries allowed adolescents to access PrEP without parental consent [56].
Social determinants	Food and housing insecurity were associated with increased HIV risk behaviours among GBMSM in the United States [57].

Several studies highlighted innovative service models which feature de‐medicalization, decentralization, diversification of options and flexible modalities, in order to increase access to prevention in vulnerable populations. In São Paulo, Brazil, people seeking PrEP or PEP have access to teleconsultations via a phone app and can pick up medication at a range of facilities, including dispensing machines and health facilities which are open 24 hours a day [52]. A randomized controlled trial assessed a phone app designed to increase HIV testing and PrEP uptake by GBMSM in Malaysia. Integration with clinical services allows users to make appointments, order testing kits and chat with clinical staff. Results demonstrated the app's potential to enhance service access and PrEP adherence [53]. These studies suggest that in settings where stigma and discrimination towards vulnerable populations are high, including in healthcare settings, mHealth has the potential to increase client autonomy.

A number of discussions focused on strategies to prevent, diagnose and treat STIs, with an emphasis on moving away from syndromic management. For example, a prospective cohort study assessed expedited therapy to primary partners among AGYW using PrEP in Johannesburg, South Africa. Among AGYW who tested positive for an STI using point‐of‐care diagnostics, 93% agreed to offer expedited therapy to their partners and 95% successfully delivered it. Reduced STI recurrence was observed, highlighting the potential of the strategy [54].

Compelling evidence demonstrated the direct impact of legal reforms and policies on HIV prevention. In Uganda, there was an alarming decrease in HIV service uptake (60%) following the enactment of the Anti‐Homosexuality Act in 2023. USAID‐supported key‐population‐led local partners responded to 2323 incidents, including evictions/forced relocations (42%), threats/discrimination (24%) and attacks/assaults (17%) on lesbian, gay, bisexual, transgender, queer and intersex people. Partners provided emergency social and legal support to address structural determinants of accessing HIV services. The experience may be relevant elsewhere given the rising number of African countries considering similar discriminatory laws or updates to criminalization codes [55]. Researchers reviewed national policies and guidelines to determine age restrictions on adolescents’ access to PrEP in Eastern, Southern, Western and Central Africa. Of the 29 identified policies, only 19 did not require parental consent for adolescents aged 12 years and above for PrEP. In many countries, there was no policy, the policy was unclear or it was misaligned with the country's HIV testing policy. The researchers recommended that laws and policies setting the age of access to HIV services should align with global norms and recommendations to not limit adolescent autonomy [56].

Several studies highlighted the contribution of social determinants such as poverty, housing and food insecurity to HIV vulnerability. In an internet survey of 9005 GBMSM in the United States, 14% reported food insecurity and 7.5% reported housing insecurity in the previous year. These insecurities were significantly associated with transactional sex, depressive symptoms, drug use and STI diagnosis. Addressing socio‐economic disparities is key to improving public health outcomes and advancing health equity [57].

## 4 DISCUSSION

In the basic science track, there was new data identifying factors promoting HIV acquisition, such as dysbiosis, and presentations describing mechanisms of HIV‐1 control by cytotoxic T lymphocytes and innate immune cells, including myeloid cells and natural killer (NK) cells. The field of HIV vaccine development continues to show promise with multiple strategies to elicit bnAb responses by vaccination advancing to clinical trials and evidence that the immunogens being tested can successfully prime naive B cells. Combination bNAbs show promise as an alternative to ARV‐based products, providing long‐acting prevention in adults and infants, and are on track to be tested in efficacy trials.

The conference showcased the promising diversity of the PrEP pipeline, most notably the data showing the superiority of twice‐yearly lenacapavir for preventing HIV compared to daily oral PrEP. A key takeaway was that “one size does not fit all”: expanded PrEP options, the development of multipurpose prevention technologies and giving clients a choice of products are essential to meet varied and dynamic preferences. The importance of involving communities and advocates at all steps of HIV research and service delivery was stressed, so that resources are invested in building PrEP product choice based on what end‐users want. We cannot just focus on new products without considering how they are delivered and the barriers faced by people who could benefit from them.

HIVR4P took place in October 2024, which may have been a high‐water mark for the field, representing the peak of achievements in HIV prevention science and of optimism around remarkable advances in HIV prevention, choice and programming. Following the inauguration of a new US president just 3 months later, the future of HIV prevention has been profoundly altered. USAID, the principal implementer of the US PEPFAR programme, has been radically reduced in scope and funding, and is now down to some 15 employees, from over 10,000 [58]. Around 90% of individuals starting PrEP globally (7.4 million people) did so through PEPFAR, including key populations, women and girls, and others at risk [59]. Under current PEPFAR policies, PrEP is only available to pregnant and lactating women at risk for HIV acquisition, a population of just a few thousand people at most [60]. This policy shift has profound implications for lenacapavir, whose rollout was planned to leverage the PEPFAR platform and infrastructure. Even if lenacapavir becomes available at low cost in low‐ and middle‐income countries through generic manufacturing, its revolutionary potential may not be realized across most of Africa, given that the programme infrastructure needed for drug distribution and clinic delivery has been so severely curtailed. HIV research supported by the United States—by far the largest funder globally through the NIH, CDC, USAID and other entities—is also facing drastic cuts. Research has been abruptly halted for transgender persons and men who have sex with men, as well as almost all US‐funded programmes in South Africa and China. This will likely blunt US‐funded innovation in HIV and has the potential to undermine the next‐generation breakthroughs needed to end HIV as a public health threat.

## 5 CONCLUSIONS

The science at HIVR4P reflected an era with more tools than ever before to prevent HIV acquisition as well as a vision of a holistic approach to HIV prevention that respects individual choice, enhances service accessibility, and is flexible to meet evolving challenges and opportunities. However, policy changes since the conference ended have profoundly altered the HIV prevention landscape and threaten the advances described in this report.

## COMPETING INTERESTS

JS, RP and VA received complimentary conference registrations from IAS. AVMS, CC, CB, DC, DD, ES, GDV, JV, KKO, L‐GB, PDR, PE, PKS, SRHT, YGh and YGa received complimentary conference registrations and limited financial support from IAS to attend HIVR4P 2024. KFO received a complimentary conference registration and limited financial support from IAS to attend HIVR4P 2024; she is supported by the US National Institute of Mental Health (R00 MH121166). DKM, EC, RM and NTL‐B received complimentary conference registrations and limited financial support from IAS to attend HIVR4P 2024, and co‐authored abstracts included in this report. BG received a complimentary conference registration and limited financial support from IAS to attend HIVR4P 2024, is the IAS President and has been a member of advisory boards for ViiV Healthcare, Merck and Gilead Sciences. MKM received complimentary conference registration, a scholarship award and limited financial support from IAS to attend HIVR4P 2024. VM‐K received complimentary conference registration, a scholarship award and limited financial support from IAS to attend HIVR4P 2024; he is a full‐time employee of IAVI.

## AUTHORS’ CONTRIBUTIONS

RP prepared the draft manuscript based on the HIVR4P 2024 conference rapporteurs’ summaries and incorporated insights from the respective presentations. The highlighted presentations were chosen by the lead rapporteurs and RP. The draft underwent multiple reviews by all co‐authors. The final manuscript is a result of their collective work.

## FUNDING

The IAS, with financial support from the US National Institutes of Health (NIH), provided funding to Roger Pebody and Dr Wendy Smith to assist with the preparation of the manuscript via an NIH grant. Other co‐authors worked on the manuscript on a voluntary basis and received only limited in‐kind support to attend HIVR4P 2024, the 5th HIV Research for Prevention Conference.

## Data Availability

The data cited is available at: https://programme2024.hivr4p.org/.
